# The Ashtavakra witnessing consciousness model: an indigenous psychological framework for transforming grief into psychological wellbeing

**DOI:** 10.3389/fpsyg.2026.1990806

**Published:** 2026-10-28

**Authors:** Kriti Vashishtha, Charu Dhankar, Shraddha Tripathi, Ruchi Joshi

**Affiliations:** Department of Psychology, Manipal University Jaipur, Dahmi Kalan, India

**Keywords:** Ashtavakra Gita, embodiment, grief, Indian Knowledge Systems, indigenous psychology, psychological well-being, witnessing consciousness

## Abstract

In modern scholarly perspectives on grieving, being saddened has been associated with bodily experience. Numbness, shaking, a heavy heart, and other physical symptoms are caused by the intense pain of loss. Most Western philosophies have increasingly focused on psychological models based on the idea of how the self adjusts to the devastating experience of losing a loved one, and this has received a lot of attention. To connect death, embodiment, self-identification, and psychological well-being, this conceptual article develops a mourning process from an Indigenous psychological perspective. This article proposes a psychological paradigm grounded in Indigenous contemplative knowledge through an interpretive analysis of the teachings of the Ashtavakra Gita. Rather than treating the Ashtavakra Gita solely as a philosophical or spiritual text, this article interprets it as an Indigenous psychological knowledge system offering culturally grounded insights into selfhood, attachment, suffering, and psychological well-being. The proposed Ashtavakra Witnessing Consciousness Model conceptualises grief as a five-stage, non-linear process involving embodied grief, existential crisis, meaning reframing, mortality acceptance, and witnessing consciousness, culminating in psychological well-being. This model shows how a traumatic incident can be transformed into a meaningful experience and a path to psychological well-being. Advaita Vedanta’s foundational text, the Ashtavakra Gita, presents a nondual viewpoint in which suffering arises from attachment to the body, ego, and interpersonal attachments. It distinguishes between witnessing consciousness and the short-lived embodied self. This psychological Model of Witnessing Consciousness offers an alternative understanding of control, attachment, suffering, and embodied grief, grounded in the Ashtavakra Gita. It highlights the psychological implications of developing the art of witnessing consciousness. This indigenous model of traditional knowledge has implications for existential, contemplative, and meaning-centred mourning therapies and adds a cross-cultural, psychological and philosophically informed perspective to bereavement research. The article explains how the five stages were derived from the text, illustrates the model with a composite case vignette, outlines clinical cautions against encouraging detachment prematurely, and sets out the model’s limitations and directions for empirical validation.

## Introduction

Contemporary theories of grief have primarily focused on the nonlinear aspects of bereavement. Through embodied, relational, and meaning-oriented views, modern grief theories have significantly improved our understanding of loss. These theories have conceptualised adaptation (after loss) through an individualistic conception of the self. For instance, Kübler-Ross's (1969) modern theory focuses on five stages of mourning that help people cope with their loss. The first stage is Denial (characterised by physical numbness and an inability to accept the loss), where the person momentarily avoids negative thoughts, giving them a window to process. In the second stage, Anger (an inadvertent way to relieve suffering), the person becomes profoundly sensitive. They attribute their losses to circumstances, other people, or themselves (loss-oriented coping). During Bargaining (the third stage), the individual tries to reverse what has happened. In this phase, they try to keep the deceased alive by sending them notes, writing to them, or posting their pictures online (as in continuing bonds theory; Klass et al., 1996). During the fourth stage, Depression (the first step in the healing process), the person begins to deal with the loss by crying and expressing grief. Lastly, Acceptance (the fifth stage), people come to terms with the loss and carry on with their daily lives. This stage aligns with Robert Neimeyer’s “Meaning Reconstruction” Theory (Neimeyer, 2001), which emphasises reconstructing the self after a loss. Neimeyer claims that following a loss, people begin to doubt their own existence. Shortly after, they discover a new life purpose and, in the end, reconstruct their sense of self in connection with the outside world and other people (restoration-oriented coping). The Dual Process Model describes these loss- and restoration-oriented processes, in which bereaved people oscillate between the two (Stroebe and Schut, 1999, 2021). Although the stage model remains popular, it has been widely criticised, and uncritical presentations of it can suggest that the stages are the only way to grieve (Avis et al., 2021). Grief has no closure in the current era. The notion of an ongoing bond holds that, although the departed is not physically present, they are mentally present for the bereaved. Instead of severing ties with the deceased, the bereaved person feels and expresses their presence, and the bond persists. Though for some it’s a healthy way to grieve (Klass et al., 1996), it still has a dual aspect as the mourner’s self-identity remains uncertain. Mourning is also linked to familiar, everyday stimuli and triggers thoughts of the deceased, thereby interfering with the griever’s well-being. Any sensory experience can trigger memories of the deceased, such as eating a dish your mother used to prepare when you were a child or listening to music from the early stages of your marriage, which can bring back memories of togetherness and love. For the mourner, there is “presence-in-absence,” as life still feels organised around the lost one’s day-to-day doings (Ter Haar and Smid, 2025). Once the grief becomes inhabited, it does not have phases that one can finish and move on from. Instead, a person learns to cope with this sadness and make it part of their existence. During the grieving process, the person learns to live life anew after the loss. This sadness remains in thoughts and feelings; it has no resolution. It significantly affects the griever’s daily activities and emotions. At times, it even impacts their sense of self and how they see the world. These modern, theory-based paradigms encourage a gradual transition from embodiment (chest heaviness, fatigue, etc.) toward emotional stability and a sense of purpose after loss (Enmalm and Boehme, 2024; Gudmundsdottir, 2009; Keisari et al., 2023; Lewis, 1988; Pearce and Komaromy, 2022).

Although grief is a universal human experience, understanding of selfhood, attachment, consciousness, and healing is culturally embedded. Contemporary grief theories have largely been developed within Western psychological traditions. These theories explain grief as somatic symptoms alongside its psychological and emotional aspects. Somatic distress has been reported across cultures and is associated with prolonged grief (Cunningham et al., 2025; Hennemann et al., 2023). Modern psychology defines psychological well-being as meaning, acceptance, self-integration, resilience, and flourishing, in addition to the absence of suffering. Indigenous traditions often view well-being as emerging from changed interactions with oneself, others, and reality, whereas Western models typically conceptualise these outcomes through individual adaptation and personal progress. In Hindu (Indian) thought, well-being rests on experiential knowledge of the self, with self-inquiry as a key facilitator, rather than on external conditions alone (Singh et al., 2023). Increasingly, scholars have argued that psychological theories should incorporate Indigenous knowledge systems that reflect diverse ways of conceptualising mental health and well-being. Indian philosophical traditions represent one such knowledge system, yet their potential contributions to contemporary grief psychology remain underexplored. Research on Indian devotional practices in bereavement is likewise limited (Joshi, 2026). To bridge this gap, the current study proposes the Ashtavakra Witnessing Consciousness Model as an Indigenous psychological framework that explains how embodied grief can become a pathway to psychological well-being through cultivating witnessing consciousness.

### The Ashtavakra Gita as an indigenous psychological and Indian knowledge system

Indigenous psychology is rooted in cultural traditions. In the Indian knowledge system, the Ashtavakra Gita is a famous Vedanta scripture. The Ashtavakra Gita does emphasise experience-based understanding (anubhava). Throughout the conversation, Ashtavakra repeatedly directs King Janaka toward immediate self-recognition rather than academic understanding. Rather than accepting something just because an instructor says it or because it’s recorded in the Vedas, Ashtavakra encourages Janaka to identify it through his own experience. The Ashtavakra Gita offers a contemporary view of embodiment and the non-dual psychology of selfhood, embodiment, attachment and consciousness. It presents a revolutionary perspective on suffering through the death of a loved one, arguing that death does not exist; it is an illusion, and the atma (self) is eternal and free from birth and death. According to Ashtavakra, one acquires knowledge by exploring one’s own thoughts, feelings, attachments, and consciousness itself. Experience, not theology, is the source of authority. The Ashtavakra Gita comprises twenty chapters in its standard recension (Ashtavakra, 1998, 2010; Rao, 2002; Stroud, 2004).

Recent literature suggests that Hindu teachings have helped Hindus cope with loss and have inculcated the idea that atma (soul) is not permanently part of the physical body, ultimately supporting detachment and healing (Shimkhada, 2023). Hindu death rituals also shape how mourners find consolation, and disrupting communal rites such as Shraddh can leave mourners without that comfort (Ghosh and Bk, 2024). The literature has not identified a distinctive contemporary Eastern model of grieving that emphasises embodiment, mortality, detachment, and the psychology of death. Furthermore, the Ashtavakra Gita is an exceptional framework that adequately explains this notion and contributes to the grieving model. Therefore, this study proposes a grief model based on the Ashtavakra Gita.

The Ashtavakra Gita distinctly separates the body and the self (atman). The body experiences sentiments, feelings, and pain, but if the self (atman) becomes the witness (sakshi) of all life’s events, the individual becomes detached and transcends physical existence. In contrast to emancipation (moksha), which occurs when the self becomes a conscious witness to all events in one’s life, pain arises when individuals attach themselves to the deceased or a lost one by identifying with the relationship.

The Ashtavakra tradition advises being aware of these pains, ageing, loss, and the changes that come with them. Through this process, one begins to recognise the embodied experiences that follow loss, gain a deeper psychological understanding of grief, and develop a stabilised self. Although we cannot completely eradicate suffering from our lives, this process increases our awareness of who we are, our strengths, and our coping mechanisms. The “Deh identification phase,” as described in the Ashtavakra Gita, is when a person experiences embodied shock upon identifying with the deceased (e.g., as a mother, son, daughter, etc.). Somatic symptoms such as heart discomfort, heaviness, numbness, emptiness, and disorientation are caused by this embodied shock. The loss seems like destruction if the self is confused with the body. This is referred to as the “ignorance” stage, in which the person exhibits physical symptoms and intense melancholy because they feel that they were unable to uphold their responsibilities to them and that their happiness was linked to the deceased since they were their parent, child, or any other family member (Chinmayananda, 2018).

The Ashtavakra Gita suggests becoming a conscious witness (Saakshi) of your own life (gains and losses) to escape this state of “ignorance.” The Ashtavakra Gita proposes that this conscious witnessing is a condition of meta-awareness in which one observes their feelings or ideas as a third party, perceiving the full picture. Instead of attributing every loss to themselves, individuals begin to see the situation more objectively and practically. Contemporary therapists use the same concept in acceptance and commitment therapy and mindfulness. Evidence for such approaches in bereavement is promising but still limited: acceptance and commitment therapy shows some benefit for accepting difficult emotions (Jones et al., 2022), and mindfulness appears to help secondary outcomes such as depression and anxiety, with mixed results for prolonged grief (Sun et al., 2025). This meta-awareness does not simply disconnect from loss; it should not be mistaken for suppressing the mourner’s sadness. Ashtavakra depicts transformation as a personal experience. The Ashtavakra Gita concludes with Janaka narrating his personal metamorphosis, unlike many philosophical writings that end with explanations. The results include changes in identity, emotions, feelings, pain, thought, behaviour, and so forth. As a result, it presents phenomenological evidence rather than empirical experiments. This interpretation approaches the Ashtavakra Gita as an existential psychology of pain, identity, and mortality rather than a religious text. Modern psychology often uses experiments to validate hypotheses. Through the psychological transformation of fears, pain, attachment, and well-being, the Ashtavakra Gita demonstrates its value. This closely parallels Indigenous knowledge systems’ experiential traditions, where efficacy is judged by lived change rather than statistical significance.

It examines how people create their identities, give their bodies purpose, and cope with transience. According to the Ashtavakra Gita, healing starts with disidentification. Its central concepts—witness consciousness, non-attachment, and liberation—come from the Indian contemplative tradition and are verified through experiential transformation rather than through externally imposed theoretical models.

Beyond being religious and focused on the Upanishads, Vedanta, Ayurveda, yoga, etc., Indian Knowledge Systems also offer a psychological stance that emphasises methodical observation of cognition, emotion, attachment, consciousness, suffering, and well-being. In Indian psychology, first-person self-observation is central to studying the mind and consciousness (Singh et al., 2023). The Ashtavakra Gita creates a cohesive view of selfhood, embodiment, attachment, mortality, consciousness and psychological well-being.

The Ashtavakra Gita asserts self-awareness via a scientific method. Identification with the deceased is the root cause of sorrow, and witnessing consciousness (a mechanism) can lead to psychological well-being and liberation. Contemporary psychology researches comparable but distinct themes.

### Re-reading embodiment, grief and psychological wellbeing through the Ashtavakra Lens

Contemporary views of grief have gradually moved away from seeing grieving as a process of separation from the departed and toward acknowledging ongoing relationships, the reconstruction of meaning, and embodied sensations of loss. By shifting the cause of grief from the loss itself to connection with the embodied self, the Ashtavakra Gita provides an alternative psychological perspective. Theories of grief are inherently vulnerable. The concerns of who is mourning, what grieving is, how to adjust and cope with loss, the healing process, how to maintain a relationship with the deceased even after death, and how to grow around sadness are all addressed by its non-linear viewpoint. The current ideas discuss the griever’s relationship with the deceased and how that relationship has impacted the griever’s emotional condition after losing a loved one. In contrast, the Ashtavakra Gita states that identifying with the deceased causes sadness. The self identifies the deceased as someone important to them and someone they relied on. When someone passes away, we feel a connection to them as a parent, child, friend, or family member; this is attachment. Additionally, the idea of having a connection to the deceased causes grief. Ashtavakra calls it “your wishes,” or the unmet desires you wished the deceased would grant. Extreme grief results from these unmet desires. Ashtavakra emphasises detachment, in contrast to contemporary views of sorrow. In the Ashtavakra Gita, detachment (asanga) means recognising that the Self is untouched by gain and loss; it shifts self-identification rather than offering a technique for managing roles. For the bereaved, this is understood psychologically as loosening possessive identification with the deceased, not as emotional indifference (Fibiger, 2015).

From the perspective of the Ashtavakra Gita, suffering arises from identification and attachment rather than bereavement alone. The goal is to overcome this connection to the objects and people in our environment. With this understanding, Janaka did not identify with his title or his roles. Although he was living in his body, he knew it wasn’t his. He understood that grief cannot be denied, but its sources could change. He learned how to be detached. Disidentification from the body, roles, and relationships allows the mourner to experience grief without being defined by it. Here, separateness is treated as phenomenologically real yet ontologically provisional (Ashtavakra, 1991).

The Ashtavakra Gita recognises that human sorrow is accompanied by physical feelings. It repeatedly attempts to distinguish between witnessing awareness, the body, and the mind. In other words, unlike modern beliefs, one may avoid decentring oneself while experiencing the bodily sensations of loss and melancholy. Ashtavakra views grieving as a process where the selfhood is not lost. According to this viewpoint, sorrow does not necessarily determine the deeper sense of self; rather, it is experienced through the body and intellect. There is no difference between grieving and repressing sadness. Instead, it is between going through pain and identifying just with the grieving experience.

People often have expectations of other people and circumstances, and when those expectations are not met, they feel depressed and begin to doubt the presence of God, their ability to survive, their aspirations in life, etc. Ashtavakra proposes equanimity, detachment, inner freedom, psychological balance, and witnessing awareness, in which we should let go of our possessions and relationships and instead show them respect and gratitude. We become upset when we try to hold on to someone because time passes, and they are no longer with us. Love that is free of possessiveness comes from detachment. Janaka’s freedom is portrayed as fearlessness and inner independence. Someone wins when they have nothing to lose. Ashtavakra philosophy emphasises that individuals are witnesses to their own consciousness, advising one to regard oneself as a visitor in a world not one’s own. Enjoy your time here as a guest; this home (the globe) is not yours, and you will peacefully depart from it when the time comes.

According to contemporary models, healing also entails rebuilding the meaning of life, maintaining a relationship with the deceased, and coping through adaptation. The self is still present here. However, according to Ashtavakra, the healing stage involves breaking down egoic attachment. An important first step is to identify grief with detachment. Examining the three layers of attachment might help dissolve egoic attachment. “Attachment with the body” (this person is no longer here), “attachment with the relationship” (this person was my partner), and “attachment with the narrative of the person” (my partner used to take care of my needs; life is nothing without them) make up these three layers (Ashtavakra, 1998).

The Ashtavakra framework examines equanimity, non-attachment, constant awareness, existential acceptance, freedom from compulsive identification, compassionate interaction with others, and inner freedom despite outward loss as aspects of psychological wellness. According to the Ashtavakra Witnessing Consciousness Model, psychological well-being is defined as a stable state of witnessing awareness, existential acceptance, non-possessive relatedness, and equanimity that results from a shift in one’s relationship with selfhood, attachment, and embodiment.

Ashtavakra does not focus on identifying selfhood with the relationships we are given in this life. People fear death because it relates to embodied grief and identifying with the deceased. People will not see death as the ultimate end of everything if they practice witnessing consciousness. When integrated, these reinterpretations suggest that mourning may be understood as a gradual shift in a person’s connection to embodiment, attachment, and selfhood, rather than merely coping with loss. The proposed Ashtavakra Witnessing Consciousness Model is conceptually grounded in these ideas.

## Theoretical foundations of Ashtavakra Gita

### Ontological foundation: self, experience, and witnessing consciousness

The Ashtavakra Gita’s description of psychological suffering and transformation can be understood through the fundamental premise that the Self is witnessing consciousness. From this viewpoint, sensations like sadness, fear, connection, and emotional pain are part of the ever-evolving field of human experience, while awareness is the entity that observes them. The Ashtavakra Gita distinguishes between identifying with pain and experiencing suffering. The subtle gap between these ideas is especially pertinent to grief, since the loss of a loved one may significantly impact one’s identity, feelings, and sense of continuity. The current framework does not interpret the ontology of the Ashtavakra Gita as an argument for rejecting or eradicating unpleasant feelings. Instead, it suggests that acknowledging a witnessing aspect of consciousness might change how a person interacts with such events. The Ashtavakra Gita describes the transition from identity-based identification (I am suffering) to experiential recognition (I am aware of suffering). This shift is ontologically supported by the ideas of witnessing awareness, non-identification, acceptance, and psychological freedom, which the suggested model explores further.

Rather than serving as a distinct psychological intervention, the Ashtavakra Gita’s ontological view of the Self provides a fundamental theoretical foundation. It establishes a sense of individuality that underpins subsequent psychological processes.

### Experiential epistemology and lived experience

The Ashtavakra Gita has a primarily experiential epistemological approach. Its main insights aim at immediate recognition or understanding of one’s own nature, rather than being offered only as ideas to be accepted intellectually. Ashtavakra and Janaka’s conversation therefore symbolises a shift from intellectual comprehension to experiential knowledge. When knowledge is encountered, analysed, and realised through one’s own experience, it becomes meaningful. Because grief is an intrinsically experienced phenomenon, this distinction matters especially. Bereavement is experienced through the body, emotions, memories, relationships, sense of identity, and existential understanding, not merely as an abstract cognitive assessment of loss. Therefore, an epistemology that engages experience as a source of psychological knowledge is necessary for a framework that aims to comprehend transformation in grieving.

Recognising the difference between temporary experiences and the awareness that observes them is directly linked to experiential knowledge in the Ashtavakra Gita. Awareness is presented as the foundation from which various experiences can be viewed, while thoughts, emotions, feelings, attachments, and situations are seen as shifting phenomena. Experience is therefore neither disregarded nor avoided; rather, it serves as the foundation for analysing the connection between the person experiencing the event, the experience, and identification with it.

### Avidya, identification, and the construction of suffering

Within this framework, suffering is not merely attributed to the occurrence of an adverse event; rather, it intensifies when transient experiences, emotions, roles, and relationships become incorporated into one’s sense of self. According to the proposed framework, avidya is more than a lack of knowledge; it is a fundamental misrecognition of the nature of the self, in which consciousness becomes identified with changing psychological and experiential contents. This provides a theoretical basis for distinguishing between grieving and identifying with grief. While the latter may sustain and escalate pain, the former is an inherent human response to loss. If association with shifting experiences maintains suffering, then acknowledging oneself as a witness to those experiences may reduce this affiliation.

### Reframing attachment and impermanence through the Ashtavakra Gita

The Ashtavakra approach examines the association between the experiencing self and the sense of loss, rather than the loss itself. Attachment is more than just an emotional connection. Suffering becomes more pronounced when people use changes in relationships, identities, roles, and experiences to define the self. As a result, the Ashtavakra Gita draws an analogy between experiencing loss and identifying with it mentally.

Attachment leads to identifying with an object or person and further distorts one’s sense of self, which in turn causes misery. The impermanence within the Ashtavakra idea of the Self contrasts transient phenomena (body, relationships, emotions, circumstances) with witnessing consciousness (the awareness in which these experiences are experienced).

### Witness consciousness (Sakshi)

Ashtavakra Gita suggests that witness consciousness is a transformative process that supports acceptance, psychological flexibility, and health by shifting the relationship between the individual and grief-related experience from identification to observation. A person’s connection with loss, suffering, and mortality may change if they go from identifying with experience to seeing it. Witnessing lessens the degree to which fleeting encounters shape a person’s entire concept of self while allowing traumatic situations to be recognised. *Sakshi*, then, is the Ashtavakra framework’s theoretical link between the experience of pain and the potential for psychological change.

### The indigenous psychological foundation of the Ashtavakra Gita: from non-attachment to psychological freedom

Acceptance and non-attachment are related processes that result from an evolving association with an experience. Experiences may still occur when identification fades, but they no longer define a person’s sense of self. In this context, non-attachment denotes a shift away from possessive or identity-based connection with fleeting events, not emotional detachment or indifference. Liberation is the release from the attachments and misidentification that sustain psychological misery. Therefore, the shift in one’s relationship with experience—where what was once seen as defining or limiting the self is increasingly viewed without full identification—is Moksha’s relevance to the current psychological paradigm. This movement aims at greater inner freedom, acceptance, composure, and adaptability.

Taken together, these ideas offer the foundation for understanding the Ashtavakra Gita as an Indigenous psychological framework. Its culturally embedded framework for understanding the self, pain, knowledge, and human flourishing gives it an indigenous identity beyond its historical Indian origins. While concepts like Sakshi (witness awareness), Asanga (non-attachment), Avidya (ignorance), and Moksha (liberation) offer culturally entrenched explanations of psychological experience and transformation, its epistemology emphasises direct experiential realisation (Anubhava). In this way, the Ashtavakra Gita provides a culturally grounded explanation of how self-knowledge, letting go of affiliation, accepting shifting experience, and moving toward psychological liberation may help reduce suffering.

## Ashtavakra witnessing consciousness model

The preceding sections laid out the philosophical and psychological roots of the Ashtavakra Gita’s conceptions of selfhood, suffering, attachment, experienced knowledge, witnessing awareness, and liberation. This section develops the Ashtavakra Witnessing Consciousness Model (AWCM), a conceptual framework for understanding the transformation of grief-related suffering, grounded in these foundations. The model is presented as a theoretically informed synthesis that translates some indigenous principles into a psychologically meaningful process of transformation, rather than a direct replication of the Ashtavakra Gita.

### Method of model derivation

The AWCM was developed through a qualitative, interpretive (hermeneutic) reading of the Ashtavakra Gita rather than through empirical data collection. We used three English translations as primary sources (Ashtavakra, 1991, 1998, 2010), supplemented by Chinmayananda's (2018) commentary. Verse numbers cited below follow the standard twenty-chapter recension. The derivation proceeded in four steps.

*Step 1: Close reading.* We read the full text and marked verses addressing the self, the body, suffering, attachment, loss, death, desire, and freedom. We set aside verses that were mainly cosmological or devotional because the aim was to identify psychologically meaningful concepts rather than summarise every teaching.

*Step 2: Concept identification.* The marked verses were grouped under recurring concepts, retained in their Sanskrit form where the text uses them: identification with the body (*dehabhimana*), the ego-sense (*ahamkara*), misrecognition of the self (*avidya*), inquiry, impermanence, the witness (*sakshi*), non-attachment (*asanga*), and liberation (*moksha*). The authors resolved disagreements about how a verse should be classified through discussion.

*Step 3: Psychological translation.* Each concept was mapped onto the closest construct in contemporary grief and well-being research. For example, we mapped identification with the body onto embodied grief, inquiry onto existential questioning and meaning-making, and the witness onto decentring and metacognitive awareness. The points at which the Ashtavakra view departs from Western models were noted at the same time.

*Step 4: Process synthesis.* The concepts were then ordered according to the movement that the dialogue itself dramatises. Janaka opens with questions about how knowledge, liberation, and dispassion are attained (1.1). He is shown that bondage lies in long identification with the body (1.14) and in the belief “I am the doer” (1.8), and that how one regards oneself shapes whether one is bound or free (1.11). He is taught that possessions and relationships are as transient as a dream (10.2) and that existence, non-existence, and change are the nature of things (11.1). He is reminded that he is the sole witness to everything, not the body or the doer (1.7; 15.4), and in the closing chapters he describes his own transformation in the first person (Chapters 19–20). Read alongside the bereavement literature, this movement yielded five clusters: immersion in embodied pain, existential questioning, re-viewing the loss, accepting impermanence and death, and witnessing. These clusters became the five stages of the AWCM. Table 1 presents, for each stage, the core concept, illustrative verses, the corresponding psychological process, and how it contrasts with existing Western models.

**Table 1:** Derivation of the AWCM stages from the Ashtavakra Gita.

Stage	Core Ashtavakra concept	Illustrative verses (paraphrased)	Psychological process	Contrast with Western models
1. Embodied Grief	Identification with the body and with roles (*dehabhimana*); the ego as a mirage	1.14: one has long been bound by the snare of identifying with the body. 1.4: resting in awareness, seeing oneself as distinct from the body, brings peace.	Somatic distress, disorientation, and loss of a role-based identity (“I was his wife”)	Somatic grief is treated as a symptom to be processed; here it also shows how far the self has merged with the body and the relationship
2. Existential Crisis	Inquiry; the ego-sense (*ahamkara*) as “I am the doer”	1.1: Janaka asks how knowledge, liberation, and dispassion are attained. 1.8: the belief “I am the doer” is like the bite of a black snake.	Questioning identity, purpose, and mortality; doubting earlier assumptions	Close to the meaning-making crisis in Neimeyer (2001), but the question turns to “Who is grieving?” and not only “Why did this happen?”
3. Meaning Reframing	The mind’s view shapes bondage and freedom	1.11: one who regards oneself as free is free, and one who regards oneself as bound is bound.	Shift from “This loss has destroyed who I am” to “This loss is part of my life but does not define me”	Meaning reconstruction rebuilds the self-narrative; here the person’s relationship to the narrative itself changes
4. Mortality Acceptance	Impermanence of all embodied conditions	10.2: friends, land, wealth, and family last like a dream or a magic show of a few days. 11.1: being, non-being, and change belong to the very nature of things.	Existential acceptance; less avoidance of reminders of death	Acceptance in Kübler-Ross (1969) concerns a particular loss; here it extends to the transience of all embodied life
5. Witnessing Consciousness	The witness (*sakshi*); non-attachment (*asanga*); liberation (*moksha*)	1.7: one is the single witness of everything and always free. 15.4: one is not the body nor the doer, but pure awareness, the witness.	Decentring, metacognitive awareness, non-reactive acceptance, and psychological well-being	Mindfulness and ACT use decentring as a technique; here witnessing is understood as recognising the nature of the self

Verses are paraphrased; numbering follows the standard twenty-chapter recension (Ashtavakra, 1998).

Two qualifications follow from this procedure. First, the Ashtavakra Gita itself points to direct, immediate recognition of the witness (*sadyomukti*) and does not describe graded stages. The five stages are therefore a psychological translation of its insights for understanding the mourner’s experience, not a ladder prescribed by the text, and witnessing may arise at any point in the process. Second, the stages are conceptual rather than temporal: a person may move forward and back between them, and they may overlap or recur (see the note to Figure 1).

**Figure 1:**
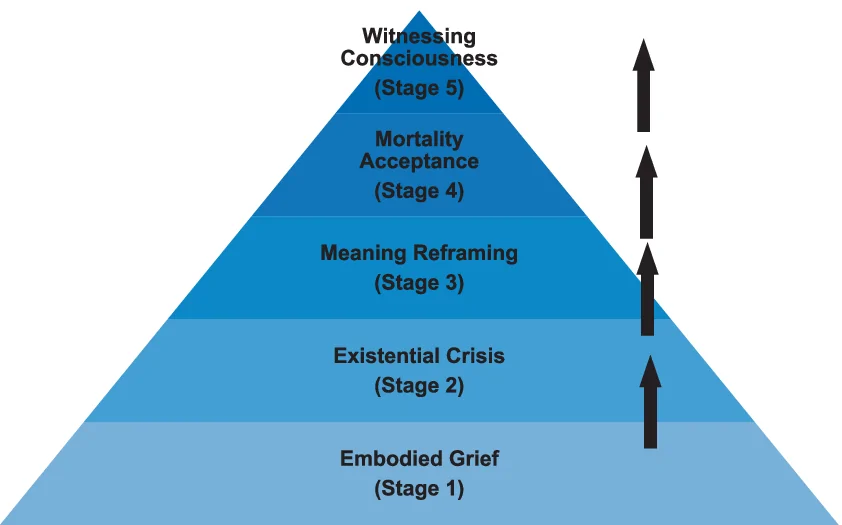
Ashtavakra Witnessing Consciousness Model for better psychological well-being. The stages are conceptual and non-linear. They may overlap, recur, or vary with each person’s experience and are not a fixed sequence.

### The five stages of the model

Most current grieving models concentrate on comprehending and reorganising the connection, processing the tremendous pain following the death of a loved one, and finding a new purpose in life to come to terms with the loss. Many individuals have found these mourning methods helpful, but they lack an existential crisis and self-identification. The Ashtavakra Gita-based psychological grief model, as suggested, emphasises the transition from subconscious distress to witnessing consciousness during the grieving process. This model comprises five non-linear, overlapping stages; Figure 1 shows the proposed Ashtavakra Witnessing Consciousness Model for better psychological well-being (Ashtavakra, 1991, 1998, 2010).

*Embodied Grief (Stage 1):* Following the loss, somatic symptoms may emerge soon. People have heartache, trembling, exhaustion, and other symptoms. The person may feel sudden emptiness, disorientation, and dysregulation. The mourner loses their sense of self due to an overwhelming attachment to the deceased. Ashtavakra calls it an illusory sense of self in Part III (Ego Mirage) of the Ashtavakra Gita. Like a physical mirage, this delusional sense of self appears genuine to the individual yet lacks an actual, independent life. According to Tajfel and Turner's (1979) social identity theory, a person’s self-identification is based on their responsibilities within relationships, group membership, nationality, occupation, etc., and it boosts their self-esteem. In Advaita Vedanta, the mind is not a blank slate (tabula rasa); it carries samskaras (latent impressions) and vasanas (deep-rooted tendencies) from past experience, and ahamkara (the ego-sense) gathers around these impressions, names, relationships, and roles to form the sense of “I.” Additionally, when someone loses a loved one, they experience certain somatic symptoms and lose their sense of direction in life because of their dependent identification with the deceased. During this stage, the person initially describes the physical and emotional aspects of mourning. Heaviness, exhaustion, sobbing, insomnia, physiological agitation, emptiness, physical tension, and so forth are a few potential signs. Empirical work similarly shows that grief sensations are felt most often in the chest, and that many bereaved people feel less present in their own bodies (Enmalm and Boehme, 2024). Although these experiences take place in the body–mind, they do not make up the whole Self. At first, the person is completely engrossed in the event. This small psychological distance can become a significant part of identity. Consequently, the individual begins to identify their stress. The therapist should not promote spiritual acceptance or detachment right away at this point. They should offer emotional safety, identify embodied distress, validate grief, and allow the individual to acknowledge their experience.

*Existential Crisis (Stage 2)*: A confrontation with mortality begins as soon as physical symptoms lessen. The bereaved person becomes obsessed with death and begins to doubt their own existence. They experience severe emotional suffering and find no meaning in daily tasks. These sentiments and beliefs have been constantly challenged by Ashtavakra. Ashtavakra asserts that one is pure consciousness beyond all of these—not the body, mind, intellect, or ego—and that the ego is a prison (Paranjpe, 2014). Those close to the departed often feel a hunger for validation after the departed’s death. During death rites, many behaviours are performed to gain society’s approval. Where to go, what to eat, how to sleep, what to do before and after cremation, etc. When such rites are disrupted, as they were during the COVID-19 lockdowns in India, mourners may lose a key source of consolation (Ghosh and Bk, 2024). All of this shows the community and family how excellent a husband, wife, son, or daughter one is, and how successfully the rites were carried out. Ashtavakra advises against trying to eradicate this ego via force. Simply become this ego’s witness, and it will disappear on its own. The bereaved starts to understand that grieving involves more than just losing a loved one; it also involves challenging preconceived notions about life. Furthermore, Ashtavakra frequently questions assumptions about possessions, existence, and selfhood.

At this level, sadness transcends emotional suffering. Existential inquiries concerning identity, purpose, security, relationships, the future, and mortality begin to arise. In this case, the mourner’s identity is disrupted because the structures they identify with become unstable. During these stages, the mourner may advance toward self-awareness, existential insight, meaning exploration, and cognitive flexibility. Rather than rushing to reduce symptoms, the therapist might create space for existential inquiry. Pushing painful emotions away at this point may prolong grief rather than relieve it (see Clinical Cautions).

*Meaning Reframing (Stage 3)*: A person begins to reframe their life’s purpose as they learn more about themselves. Ashtavakra addresses changing viewpoint, in contrast to classic theories of meaning reframing, in which the bereaved either maintain their connection to the departed or divert themselves through other activities. According to current theories, the mourner attempts to reframe the significance of past events and their relationships through persistent cognitive and existential reappraisal. Ashtavakra, on the other hand, suggests understanding what has transpired. The bereaved person should reflect on their life and realise that change is a constant. They should observe that transformation. The body, mind, and thoughts are ever-changing. Therefore, it’s critical to acknowledge that we are only observers and not the body, mind, or ideas. From this viewpoint, one can look beyond one’s ego and enter a self-trance. This stage links pain and acceptance. When the ego fades, we are left with a real sense of self, true tranquillity, true pleasure, true temperament, and true nature. Ashtavakra calls that the “real self.”

Self-reflection, non-identification, and altering one’s relationship with experience result from reframing the relationship between the person and the loss. The person starts to change from “This loss has destroyed who I am” to saying, “This loss is an experience within my life, but it does not completely define who I am.” This shift may lead to psychological flexibility, emotional control, meaning reconstruction, and less identification with suffering. During this period, clinical treatments can support meaning-making, perspective-taking, narrative reconstruction, and reflective inquiry.

*Mortality Acceptance (Stage 4)*: At this point, the bereaved person acknowledges that death is unavoidable. This is more than accepting loss; it’s a global shift in perspective: nothing lasts forever, and attachment cannot avoid loss. By referring to the body and mind as resting places that are not one’s own, Ashtavakra lessens the anxiety of death. After experiencing wins and losses while residing in the body, we eventually depart from both the body and the mind. The soul is only a traveller. People act in life for society’s approval and others’ happiness, not realising that this place of rest is only transitory. However, Ashtavakra believes that we must focus on finding the eternal resting place inside. For a while, the bereaved person recalls pleasant experiences with the deceased. These recollections evoke different emotions, but they eventually begin to fade. People come to an existential acceptance of this reality.

This stage does not imply putting an end to grief by accepting death. Here, acceptance is recognising the transience, loss, death, and unpredictability of some life situations. The Ashtavakra idea of embodied existence’s evolving character and the difference between fleeting events and witnessing consciousness are closely related to this stage. “This should not have happened.” eventually gives way to “This has happened, and I can acknowledge its reality without being completely consumed by it.” Here, the person comes to embrace psychological flexibility, emotional regulation, and existential acceptance. Additionally, they avoid certain situations less.

*Witnessing Consciousness (Stage 5):* A child is carefree, happy, and disconnected at birth, but over time, their name, relationships, roles, responsibilities, and identity become tied to them. This forms the foundation of the “I” (ego), and the individual works throughout life to protect the “I” they are building. Maybe we will be able to live better if we acknowledge this and come to terms with the fact that death is unavoidable. After coming to this realisation, the bereaved person begins dedicating themselves to helping others, forming more meaningful relationships, and leading a more rewarding life every day. In the end, this results in post-traumatic recovery and personal development. While eudaimonic well-being comes from meaning-centred coping, the ego makes it personal. We are more aware of our surroundings when we live in the now and do not worry about the past or the future. Ashtavakra advises observing from a third-person perspective, letting thoughts flow, and staying in a state of flow. Enjoy the flow state by being aware of your own surroundings rather than forcing yourself to think positively.

This stage examines how one’s connection with sorrow has changed. Acknowledging awareness’s ability to see thoughts, feelings, and experiences without fully identifying with them is a key component of witnessing consciousness. This is the fundamental indigenous concept. From a psychological perspective, the person shifts from “suffering” to “awareness of that suffering.” At this point, the therapeutic activity may foster reduced identification, non-reactive awareness, acceptance, metacognitive consciousness, and reflective observation. By this stage, the person experiences improved psychological well-being, and this promotes existential acceptance, psychological flexibility, inner freedom, and emotional regulation.

The Ashtavakra Gita mentions three key processes that led to this concept. These processes include awareness (recognising ideas and feelings without identification), inquiry (questions about oneself, relationships, death, and permanence), and reorientation (reframing life’s purpose and shifting viewpoint). The suggested model does not view sorrow as a pathology to be treated or a series of emotional phases to be completed. Instead, it draws on the Ashtavakra Gita and views grief as an existential process that can deepen understanding of death, meaning, and purposeful existence through embodied sorrow. In this way, grieving may lead to new self-knowledge and psychological well-being rather than simply being a reaction to loss.

### Illustrative application: a composite case vignette

The following vignette is a hypothetical composite written by the authors to show how the model might guide practice. It does not describe a real client, and the stages are presented in order only for clarity.

Meera (a pseudonym), a 54-year-old schoolteacher, lost her husband suddenly to a heart attack. In the first weeks, she reported a heavy chest, sleeplessness, and a feeling that “half of me has gone.” She described herself almost entirely through the relationship: “I was his wife for thirty years; now I do not know who I am” (Stage 1). In keeping with the model, the counsellor did not introduce any idea of detachment. Sessions focused on safety and sleep, naming bodily sensations, and giving her room to cry and talk about her husband.

After the thirteenth-day rites, Meera began to ask, “Why him? What is the point of my routine now? What happens after death?” Relatives also judged her on whether she had performed the rituals correctly (Stage 2). The counsellor treated these questions as meaningful rather than symptoms to be reduced and invited her to reflect on a question from the text: “Who is the one who is grieving?”

Over the following months, Meera moved between intense sorrow and moments of reflection. She gradually began to say, “Losing him has changed my life, but it is not the whole of me” (Stage 3). Narrative work helped her to honour the marriage while recognising other parts of her identity as a teacher, a mother, and a friend. Around the first death anniversary, she spoke of a new awareness that “everything we hold is only for a while,” and her fears about her own death and her children’s safety began to ease (Stage 4).

With her consent, the therapist then introduced brief practices of observing thoughts and bodily sensations. Meera reported that waves of sadness still came, but that she could “watch them come and go” without feeling destroyed by them (Stage 5). A family crisis later returned her to acute distress and bodily symptoms, illustrating the non-linear movement the model proposes, and the counsellor returned to the stabilising work of Stage 1. Throughout, she was screened for prolonged grief and depression, with referral to a psychiatrist available if needed.

The vignette shows the practical order the model implies: validation before reflection, inquiry before reframing, and witnessing offered only when the person is ready, without assuming that grief and well-being cannot coexist.

### Implication of the Ashtavakra witnessing consciousness model

This model expands current theories of grieving. It offers a novel viewpoint on detachment, awareness, and mortality. It offers an alternative way of identifying with the self. The Ashtavakra Gita discusses embodied grief from several ideological perspectives. It explores grief from both embodied and existential perspectives. It underscores the role of attachment and identification in grief, helping the mourner transform through acceptance of mortality. Rather than only creating meaning, the healing process emphasises reframing meaning. According to Ashtavakra, witnessing may alter a person’s perspective on bereavement. Rather than if wellness entails the eradication of sadness, the paradigm permits grief and wellbeing to coexist. The goal is a transformed relationship with grief, not the absence of sadness.

The Ashtavakra Witnessing Consciousness Model presents an alternative ontological foundation. Its distinctive claim is that grief intensifies through identification with what has been lost, rather than arising from loss alone. Understanding this viewpoint is crucial for healing and detachment (a deeper comprehension of selfhood). This model focuses on the nondual relationship between sadness and self-identification. The mourner experiences this painful journey rather than living in denial or diverting their attention from their loss. As an observer, they ought to experience the agony and the embodied bodily sensations. The frequency and severity of the discomfort should be their main concerns. After that, the person experiences happiness and misery on a single level; nothing significantly affects them. The individual is no longer reliant on others and society to validate their emotions. According to the suggested model, grieving is a dynamic transition between reflective knowledge of physical experience and physiological immersion in loss. By examining how experiences of loss may modify basic conceptions of selfhood and corporeality, this viewpoint broadens the scope of contemporary embodiment studies.

Theoretical frameworks from many philosophical and cultural traditions are necessary to globalise grief studies. The Ashtavakra Gita offers an alternative conception of death and attachment, a nondual notion of selfhood, and a South Asian perspective (Ashtavakra, 2010). This model adds to the diversity of mourning studies by offering a framework that is both psychologically and culturally relevant. Recent work on Hindu death rituals and on devotional coping among Indian widows likewise shows the value of culturally grounded approaches to bereavement (Ghosh and Bk, 2024; Joshi, 2026).

### Clinical cautions

The Ashtavakra Witnessing Consciousness Model is a conceptual framework and has not yet been tested empirically; it should not be treated as a validated intervention. Because the Ashtavakra Gita teaches non-attachment in a radical, nondual form, apply its ideas with care in clinical and counselling settings. Therapists should not encourage detachment prematurely, particularly when a person is experiencing intense grief or distress. The bereaved person should first be given adequate space to acknowledge and process the immediate physical and emotional experiences of grief (Stage 1) before deeper practices of witnessing or non-attachment are introduced.

Introduced too early, or understood as an instruction to feel less, detachment may be experienced as pressure to suppress grief and can result in emotional numbness, dissociation, or avoidance. Avoidance-based coping is itself a risk factor for prolonged grief (Eisma and Stroebe, 2021), and contemplative practices can occasionally have adverse effects in vulnerable individuals, which has prompted calls for trauma-sensitive adaptations (Sun et al., 2025). Witnessing awareness, as described in this model, is not a way to avoid or suppress grief; it follows acknowledgement of loss and does not replace it.

Practitioners are advised to follow the person’s own pace and readiness; to validate and normalise bodily and emotional reactions first; to monitor for signs of numbing, dissociation, or worsening distress and to pause any witnessing practice if these appear; to screen for prolonged grief, depression, and risk of self-harm and refer to qualified mental health professionals where needed; and to respect the person’s own cultural and spiritual beliefs without imposing a philosophical position. The model is intended to complement, not replace, established bereavement care.

### Limitations of the model

Several limitations should be acknowledged. First, the model is derived from the interpretation of a single text. The five stages translate a nondual scripture, which describes immediate recognition of the Self, into a psychological process; they reflect the authors’ reading of the cited translations and commentaries, not empirical data. Scholars of Advaita Vedanta may weigh the verses differently, and some nuance is inevitably lost when Sanskrit concepts such as *sakshi* or *asanga* are rendered in English and in psychological terms.

Second, the model rests on nondual metaphysical premises, such as the claim that the Self is untouched by birth and death, which cannot be tested empirically. What can be tested are its psychological claims: that reduced identification with the loss, greater decentering, and acceptance of impermanence are associated with lower grief-related distress and higher well-being. Third, several of these processes overlap with existing constructs such as decentering, self-as-context in acceptance and commitment therapy, and nonattachment (Fresco et al., 2007; Sahdra et al., 2010). Future work will need to show what the model adds beyond these constructs, for example, in its account of identification with roles and relationships and its emphasis on mortality acceptance.

Fourth, the model draws on a Hindu philosophical tradition and was developed with Indian mourners in mind. Its relevance for people of other faiths, for secular clients, and for Indian clients who do not relate to Vedantic ideas cannot be assumed. Fifth, it describes adult bereavement in general and does not yet address traumatic or violent deaths, the death of a child, bereaved children and adolescents, or prolonged grief disorder, each of which may require specialised care. Finally, the comparison with Western theories has focused on a small number of influential models (Kübler-Ross, 1969; Neimeyer, 2001; Stroebe and Schut, 1999) and does not cover the full range of contemporary grief research.

### Directions for empirical validation and clinical application

The model generates testable propositions, and empirical work could proceed in four phases. (a) *Content validation:* a Delphi study with scholars of Advaita Vedanta, grief therapists, and clinical psychologists could assess whether the stages faithfully represent the text and are clinically meaningful. (b) *Qualitative exploration:* interpretative phenomenological or reflexive thematic analyses of interviews with bereaved Indian adults could examine whether people recognise the stages in their own experience, how they move between them, and whether witnessing emerges spontaneously or only with guidance.

(c) *Measurement and longitudinal testing:* the model’s constructs can be operationalised with established instruments, such as the PG-13-Revised for prolonged grief (Prigerson et al., 2021), the Experiences Questionnaire for decentring (Fresco et al., 2007), the Nonattachment Scale (Sahdra et al., 2010), and Ryff's (1989) scales of psychological well-being, alongside a new measure of the AWCM stages. Longitudinal designs could test whether decentring and nonattachment predict later reductions in grief severity and gains in well-being, and could map the non-linear movement between stages. (d) *Intervention studies:* a manualised, culturally adapted individual or group programme based on the model could first be tested for feasibility, acceptability, and safety, including monitoring for adverse effects, and then compared with standard bereavement counselling in a randomised controlled trial. Mediation analyses could test whether changes in decentring and nonattachment explain changes in outcomes.

In clinical settings, the model may be most useful as a complement to existing bereavement care in Indian hospices, palliative care units, and community counselling services, and as a framework for training counsellors to respond to the existential and spiritual questions that clients raise after a loss. Any such use should follow the cautions described above.

## Conclusion

The model’s main contribution is the idea that a culturally grounded shift in the individual’s relationship with experience—from identification and immersion toward awareness, inquiry, acceptance, and witnessing—may change grief-related suffering, rather than the assertion that grief follows five set stages.

Ashtavakra asserts that once we master the skill of witnessing awareness, we make the most of every moment, mindful that it will soon depart. We may be alone but not lonely. There are no regrets, tension, or worry in life. When a loved one passes away, people are afraid of that void since it is connected to their identity. However, one should take pleasure in one’s own company. The intensity of events that cause us anguish diminishes when we observe emotional suffering and negative thoughts without comparison or judgment. By avoiding mental conflict, Ashtavakra teaches us to let go and enter a state of flow. Our brains should default to peace.

A delusion of control exists in those who cannot examine their present. And this belief causes anxiety, tension, and depression. The science of surrender is taught in the Ashtavakra Gita (be in a state of flow, see everything from a third-person perspective, and find serenity within). Be the witness of your consciousness rather than allowing the loss of someone to become a permanent part of your life.

## Data Availability

The original contributions presented in the study are included in the article/supplementary material, further inquiries can be directed to the corresponding author.
